# Book Reviews

**Published:** 1892-09

**Authors:** 


### *BOOK REVIEWS.*

---

New Pronouncing Dictionary of Medicine. By John M. Keating, M. D., LL. D., Fellow of the College of Physicians of Philadelphia; Vice-President of the American Pediatric Society; editor "Cyclopedia of the Diseases of Children," etc; and Henry Hamilton, author of "Translation of Virgil's Æneid into English Rhyme," etc., with the collaboration of J. C. DaCosta, M. D., and Frederick A. Packard, M. D. W. B. Saunders, Philadelphia.

Authors and publishers have furnished us with many medical dictionaries within the last three years. Between the small and convenient volume of Gould and the immense and exhaustive work of Foster and his army of collaborators there is a great void, which has not been occupied since the classical work of Dunglison went out of date. An attempt to do this is seen in the volume now before us. The authors have given us a one-volume dictionary of 818 pages, with the definition, derivation and pronunciation of scientific and medical words. The pronunciations of certain words, while correct, doubtless, are in the nature of innovations and will not be adopted in a hurry. The definitions are accurate and condensed, and no useless matter has been included. A useful appendix concludes the volume, containing tables of weights and measures, classifications of pathogenic and non-pathogenic bacteria, tables of poisons and antidotes, list of the newer drugs, with their properties and therapeutic indications, etc. The table of diseases, designated by the names of their discoverers, will be useful, as long as the tendency exists which associates a disease with the name of the observer who first described it. It is not everybody that knows what *Morrand's foot* is, or *Trousseau's phenomenon.*

We believe that this book is the best one-volume dictionary now accessible, and there is no doubt that it will gain popular acceptance.

TRANSACTIONS OF THE SOUTHERN SURGICAL AND GYNECOLOGICAL ASSOCIATION. VOL. IV. Dr. W. E. B. Davis, Secretary, Rome, Ga.

The present volume is the report of the transactions of the meeting held in Richmond. This Association, now four years old, has done some excellent work and contributed much valuable material to the literature of medicine. In point of scientific interest and merit the papers published in this volume seem to us to be an advance of those which have been issued heretofore by the Association. A small objection might be raised that the *gynecological* element predominates. To the able and enthusiastic secretary, Dr. Davis, the thanks of the Association are due for the zeal and efficiency which he has shown in his work.

Some of the more important papers are: The President's address, "A Plea for Progressive Surgery," by Dr. L. S. McMurtry, of Louisville; "Complications in Pelvic and Abdominal Surgery," by Dr. Joseph Price, of Philadelphia; "Removal of Necrotic and Carious Bone with Hydrochloric Acid and Pepsin," by Dr. Robt. T. Morris, of New York; "The New Field of Abdominal Hysterectomy," by Dr. J. F. W. Ross, of Toronto; "Some Complications of Psoas Abscess," by Dr. J. McF. Gaston, of Atlanta; "A Case of Cholecystotomy for Stones in the Gall-Bladder," by Dr. W. E. B. Davis, of Rome, Ga.

---

A DICTIONARY OF TREATMENT, OR THERAPEUTIC INDEX. By William Whitla, M. D., Professor of Materia Medica and Therapeutics in the Queen's College, Belfast; author of Pharmacy, Materia Medica and Therapeutics, etc. Lea Bros. & Co., Philadelphia.

This is one of the most useful works which the physician can place in his library. It is a practical treatise on both medical and surgial therapeutics, and the alphabetical arrangement of subjects makes it convenient as a work of ready reference. Portions of the work that are worthy of special notice are those describing the treatment of diabetes, the heart diseases, and typhoid fever. The author still retains the obsolescent names, typhlitis and perityphlitis. Under these titles we find him advocating a policy of what may be called apprehensive inactivity. We are somewhat surprised that he justifies the use of the hypodermic needle to establish the existence of pus.

Those who read this book will find almost every disease well discussed and its treatment well outlined. By those who have it it will be used often, and generally, we think, with entire satisfaction.

---

DISEASES OF THE NERVOUS SYSTEM. By Jerome K. Baudny, M. D., LL. D., Professor of Diseases of the Mind and Nervous System, Missouri Medical College, St. Louis; Late Physician-in-chief to St. Vincent's Institution for the Insane, etc. Second Edition. J. B. Lippincott Company, Philadelphia.

This book is a publication of the author's lectures to students. It is a fair class-room description of the diseases of the nervous system, with their symptoms and treatment, etc., but contains no new matter or anything to which the reviewer may call especial notice. The writer, or lecturer, does not hesitate to make free and frequent quotations from other authors. *Insanity* in its several relations is very excellently discussed, and these lectures constitute really the best portion of the work.

---

DISEASES OF THE URINARY APPARATUS. By John W. S. GOULEY, M. D., Senior Surgeon to Bellevue Hospital, New York. D. Appleton & Co., New York.

In this work the eminent author has dealt exhaustively with the phlegmasic affection of the urinary apparatus.

The book is well written and evidences the care and research expended in its compilation. The treatise appeals more to the practitioner than the student, and contains the latest advances in the therapeutic art as applicable to genito-urinary medication.

The extended experience, and deserved reputation of the writer must carry great weight in causing the acceptance of his deductions, and his clear, concise and logical presentation of the same will entitle his book to an appreciative reading by many.

